# A biopsychological network approach to variables contributing to preoperative quality of life in patients undergoing cardiac surgery

**DOI:** 10.1038/s41598-025-93467-7

**Published:** 2025-03-13

**Authors:** Stefan Salzmann, Kilian Stenzel, Monika Sadlonova, Frank Euteneuer, Nicole Horn, Ardawan J. Rastan, Juliane Lenz, Andreas Böning, Miriam Salzmann-Djufri, Bernd Niemann, Meike Shedden-Mora, Johannes A. C. Laferton, Winfried Rief, Max Berg

**Affiliations:** 1 Department of Clinical Psychology and Psychotherapy, Philipps University of Marburg, Marburg, Germany; 2 Medical Psychology, Department of Medicine, Health and Medical University, Erfurt, Germany; 3 Department of Psychosomatic Medicine and Psychotherapy, University Medical Center Göttingen, Göttingen, Germany; 4 Department of Cardiovascular and Thoracic Surgery, University Medical Center Göttingen, Göttingen, Germany; 5 Department of Psychiatry, Massachusetts General Hospital, Boston, USA; 6 German Center for Cardiovascular Research (DZHK), Partner Site Göttingen, Göttingen, Germany; 7 Faculty of Human Sciences, Division of Biological Psychology and Neuroscience, Vinzenz Pallotti University, Vallendar, Germany; 8 Department of Cardiovascular Surgery, Heart Center, Philipps University of Marburg, Marburg, Germany; 9 Department of Cardiovascular Surgery, Justus Liebig University, Giessen, Germany; 10 Department of Psychology, Medical School Hamburg, Hamburg, Germany; 11 Department of Psychosomatic Medicine and Psychotherapy, University Medical Center Hamburg- Eppendorf, Hamburg, Germany; 12 Institute for Mental Health and Behavioral Medicine, Department of Medicine, HMU Health and Medical University Potsdam, Potsdam, Germany; 13 Department of Clinical Psychology and Psychotherapy, Philipps University of Marburg, Gutenbergstraße 18, 35032 Marburg, Germany

**Keywords:** Network analysis, Quality of life, Expectations, Biopsychosocial, Cardiac surgery, Psychology, Cardiology

## Abstract

Quality of life (QoL) in cardiac surgery patients is increasingly recognized as a critical outcome, influenced by biopsychosocial variables. This study aims to explore the associations between preoperative QoL and various psychological and biomedical variables in patients undergoing cardiac surgery. The study includes cross-sectional baseline data from 204 cardiac surgery patients in two distinct cardiac surgery samples: Data collection for the PSY-HEART I trial (coronary artery bypass grafting) was conducted from 2011 to 2015, while data for the ValvEx (valvular surgery) trial were collected between 2020 and 2022. We assessed psychological variables, such as illness beliefs and expectations, alongside biomedical variables, including body mass index, EuroSCORE II, and C-reactive protein levels. Data analysis involved partial correlation Gaussian Graphical Models (GGM) and Directed Acyclic Graphs (DAGs) to identify key nodes and pathways affecting QoL. The resulting GGM was estimated to be rather sparse (38 of 136 possible edges were present) and the case-drop bootstrap node stability estimates ranged from sufficient (CS-Coefficient Bridge Expected Influence = 0.28) to good (CS-Coefficient Expected Influence = 0.51). Our analyses revealed strong associations between psychological variables and preoperative QoL, with current and expected illness-related disability being central to the network. Medical variables showed weaker connections to QoL. The DAG indicated that expected disability influenced current disability and preoperative QoL, suggesting that preoperative expectations may be crucial for postoperative outcomes. This study underscores the importance of psychological variables, particularly illness perceptions and expectations, in determining QoL in cardiac surgery patients. Targeting these variables through preoperative interventions may enhance postoperative recovery and QoL, advocating for a biopsychosocial approach in cardiac surgery care.

**Supplementary Information:**

The online version contains supplementary material available at 10.1038/s41598-025-93467-7.

## Introduction

Besides mortality and morbidity, patient reported quality of life (QoL) has become a central outcome measure in cardiac surgery^1^. Despite significant advancements in surgical techniques that have markedly improved outcomes, the postoperative QoL often continues to be impaired even when the procedure is considered medically successful^2,3^. Numerous studies have demonstrated that patients with cardiac diseases experience substantial psychological distress, which may adversely affect their QoL^4,5^. Research also indicates that psychological factors, such as patients’ expectations and illness beliefs, significantly influence postoperative outcomes such as patients’ QoL independent of medical risk factors^6,7^. Consequently, for providing patients with the best healthcare possible, focusing solely on refining surgical techniques and addressing biomedical risk factors is insufficient. An integrated approach based on a biopsychosocial model considering biomedical, psychological and social aspects and their respective interactions is essential to enhance patient outcomes^8,9^. However, existing research commonly focuses on a small number of isolated variables per study, thereby neglecting the complex interplay of different variables. This work aims to evaluate the associations between patients’ preoperative QoL and various psychological (e.g., treatment and illness expectations, perceived illness-related disability) and biomedical variables (e.g., mortality risk scores, body mass index [BMI], and systemic inflammation as indexed by C-reactive protein [CRP]) in cardiac surgery patients.

Inflammatory markers such as CRP, BMI and risk scores predict future cardiovascular events and mortality in several populations including patients undergoing cardiac surgery^10–13^. However, variables predicting mortality do not necessarily predict QoL after cardiac surgery^14^. Therefore, other variables such as patients’ expectations may also be of relevance^7^. Expectations, as a central mechanism driving placebo effects, contribute substantially to clinical outcomes in various fields of medicine^15,16^. The non-specific effects of surgery (the effects that are due to placebo effects and not specific effects of the surgery) are generally considered large^17^. Consequently, expectations about recovery can influence patients’ perceptions and reports of their health status, adherence to rehabilitation protocols, and overall satisfaction with the surgical procedure^18,19^. Positive expectations are often associated with better recovery trajectories, while negative expectations can lead to increased anxiety, poor adherence to medical advice, and worse health outcomes^19^. Similarly, illness beliefs – which encompass patients’ perceptions and understanding of their condition – can affect their emotional well-being, coping strategies, and engagement with postoperative care^20,21^. Patients who perceive their illness as a severe and uncontrollable threat may experience elevated levels of stress and helplessness, hindering recovery^19,20^. In contrast, those with a more optimistic and manageable view of their illness are likely to engage more actively in their recovery process, leading to better physical and psychological outcomes. Addressing and modifying patients’ expectations and illness beliefs through targeted interventions may significantly enhance postoperative recovery and overall QoL. However, considering the high number of different kinds of expectations^22^, it is challenging to decide which expectations should be targeted.

Network analyses offer interesting methods for understanding the complex interplay between various variables influencing postoperative outcomes. Unlike traditional approaches that often examine single or a few isolated variables, network analyses enable researchers to explore the intricate web of relationships among multiple psychological, social, and biomedical variables simultaneously^23,24^. This holistic approach can identify key nodes and pathways within the network that significantly impact patients’ QoL. By mapping out these connections, network analyses may uncover previously hidden patterns and unique interactions, providing a more comprehensive understanding of how different factors contribute to postoperative recovery. Additionally, this method can help identify potential targets for intervention, enabling the development of more effective personalized treatment strategies. By using network analyses, researchers can move beyond simplistic models and embrace a more integrated perspective, ultimately leading to improved patient outcomes and a deeper insight into the biopsychological determinants of health.

This research examines how medical and psychological variables interact with patients’ QoL before undergoing cardiac surgery by employing different network analyses. The variables in this study were selected for their established relevance to QoL outcomes in cardiac surgery patients, focusing on psychological and medical domains. Psychological variables, such as illness perceptions and expectations, were included due to their significant influence on recovery, emotional well-being, and postoperative disability. Medical variables, including C-reactive protein (CRP), European System for Cardiac Operative Risk Evaluation (EuroSCORE II), and body mass index (BMI), were chosen as key indicators of physical health and surgical risk. A better understanding of the variables associated with preoperative QoL is important, since patients’ QoL prior to cardiac surgery is predictive of QoL after surgery^25^. Identifying the most influential variables associated with QoL may enhance opportunities for prehabilitation - preparing patients optimally before surgery to optimize postoperative outcomes^26,27^. This research seeks to bridge the gap between studies on isolated variables and a more integrated biopsychological approach, ultimately contributing to the development of more holistic and effective interventions targeting the most relevant variables for improving patient QoL before and after cardiac surgery. To summarize, this study aimed to address the following research question: How do psychological variables (e.g., perceived illness-related disability, and illness perceptions and expectations) and biomedical variables interact within a network structure and how are they associated with preoperative quality of life in patients undergoing cardiac surgery?

## Methods

### Participants

This work presents cross-sectional baseline data from cardiac surgery patients in two distinct cardiac surgery samples: The first sample consisted of 115 patients from the PSY-HEART I trial^28^, scheduled for coronary artery bypass graft (CABG) surgery with or without valvular surgery. The second sample included 89 patients from the ValvEx study (Horn et al., 2025, accepted), scheduled to undergo valvular surgery. Data collection for the PSY-HEART I trial was conducted from 2011 to 2015, while data for the ValvEx trial were collected between 2020 and 2022. The inclusion criteria for both samples were that it had to be the patients’ first elective cardiac surgery, and that cardiac disease had to be the primary source of their perceived disability. All patients included in the study were informed both verbally and in writing and provided verbal and written informed consent. Since network analysis methods typically require sufficient large sample sizes, we combined both datasets to ensure reliable edge detection and model stability. The analysis focused on preoperative QoL rather than postoperative QoL because the data were derived from two RCTs, making it challenging to disentangle the effects of the interventions applied in these studies. Additionally, the postoperative assessments had a higher percentage of missing data, further limiting their use.

### Measures

We included measures that were assessed in both samples. The following psychological variables as indicators of the current illness burden and QoL were assessed: a modified version of the Pain Disability Index (PDI)^29^, tailored to patients undergoing cardiac surgery. This scale evaluates disability across seven life domains (e.g., family, job, and social activities), with ratings ranging from 0 to 10 (higher scores indicating higher disability), reflecting the impact of a patients’ most important health issue. It allows for comparison with general population data and generates a total disability score^30^. QoL was measured using the Short Form Health Survey 12 (SF-12) version 1.0^31^ which is one of the most frequently used assessment tools for QoL allowing to assess a physical and mental QoL (PH, MH) subscale. For assessing patients’ illness beliefs, the Brief Illness Perception Questionnaire (B-IPQ)^32^ as a widely used 9-item questionnaire was applied; since item 9 of this questionnaire asks for qualitative answers, we excluded it from further analysis. Each of the following domains is assessed with one item each: consequences—impact of the disease on one’s life (ICO), timeline-acute/chronic—perception of disease duration (ITL), personal control—control over the disease (IPC), treatment control—perception of the treatment’s impact (ITC), identity—symptoms experienced (IID), illness concern—worry about the disease (later indexed in ICR), illness coherence—understanding of the disease (ICH), emotional representations—emotional effect of the disease (later indexed in ICR), and cause—perceived cause of the disease (qualitative item).

Patient expectations were assessed using the following measures: First, patients’ expected disability due to their cardiac disease six months after surgery was measured using the items of the PDI (see above) regarding daily living (EXD). Second, patients’ expectations were further evaluated using subscales from the Expected Illness Perception Questionnaire, which is derived from the Illness Perception Questionnaire^33^. Treatment expectations were gauged by three items from the “treatment control subscale” (EXT), such as “Six months after CABG surgery, the surgery has cured my coronary disease.”; personal control expectations (EXP) were assessed through four items from the “personal control subscale”, such as “Six months after CABG surgery, there is a lot I can do myself to control my symptoms.”; expected consequences (EXC) were assessed using the four items of the expected consequences subscale, such as “Six months after my cardiac surgery, my cardiac disease will have significant impacts on my life.“.

For medical variables, we utilized the European System for Cardiac Operative Risk Evaluation (EuroSCORE II, EUR)^34^ as a risk marker for 30-day mortality after surgery (calculated by adding scores from several risk factors such as age, sex, chronic pulmonary disease, diabetes, serum creatinine and left ventricular dysfunction), BMI, and inflammation as indexed by hs-CRP, which has been shown to predict worse immediate^35^ and long-term outcomes^36–38^ after CABG surgery. The assays had a sensitivity of 0.02 µg/ml for CRP. Since age and sex are already integral components of the EuroSCORE II calculation, we refrained from including them as separate additional parameters in the analysis to avoid topological overlap. Additionally, education was not considered in the analyses due to its dichotomous nature and the lack of a larger sample size required to test its potential moderating effects.

Baseline assessments in the PSY-HEART I trial were conducted approximately seven days before surgery, whereas in the ValvEx study, baseline assessments took place one day prior to surgery. For more details regarding methodological aspects or outcomes, please refer to the respective PSY-HEART I^28^ and ValvEx^39^ papers.

### Data analysis

#### Data Preparation and preliminary analyses

The analyses were performed on 204 patients (*n* = 115 from the PSY-HEART trial, *n* = 89 from the ValvEx trial). We employed single-point imputation using the expectation-maximization algorithm to address missing data, providing unbiased parameter estimates and improved statistical power when the amount of missing data is low and missing completely at random^40,41^. Our dataset met these criteria, as evidenced by a non-significant result from Little’s Missing Completely at Random (MCAR) test (Chi-Square = 604.145, df = 570, *p* = .156) confirming that the missings occurred at random and less than 3.5% missing values are present in the dataset.

Due to non-normal distribution, the variable “CRP” was transformed using the natural logarithm to obtain a normal distribution. Since the variable “EuroSCORE II” violated the benchmarks of skewness > |2| and kurtosis |7^42^, we used nonparanormal transformation using the R package “huge”^43^ to all variables according to common recommendations for network analyses^44^.

To investigate potential topological overlap (redundant measurement of the same underlying construct) of variable pairs, we used an established data-driven procedure^45^. First, we tested if our correlation matrix was positive definite (all eigenvalues > 0, invertibility). Then, we searched for highly inter-correlated variable pairs (*r* > .50) that also correlated to the same degree with other variables (i.e., < 25% of correlations are significantly different for a given pair) using the goldbricker function of the R package networktools^46^. This method suggested reducing the items 6 (“concern”) and 8 (“emotional response”) from the BIPQ, as they were only significantly different in 6.25% of cases. Due to the subjective^47^ and objective^48^ centrality of concern (i.e., rumination and worry) in network studies on psychopathology we decided to average the items to receive an index of emotional burden.

We present a visualization of the data distributions after standardization and after nonparanormal transformation (Figures S1 A and B) using raincloud plots^49^ with the help of the ggrain package in R. Furthermore, we present the correlation matrix of the items after preprocessing (after item averaging and nonparanormal transformation).

### Gaussian graphical model

We performed a cross-sectional network analysis using a Gaussian Graphical Model (GGM). In these models, a thicker edge indicates a larger partial correlation between two nodes, with red color indicating negative and blue color indicating positive associations. The edges represent unique connections between two nodes, with all other correlations in the network have been partialized out. According to the node placement algorithm used^50^, nodes that are more strongly correlated appear in close proximity.

The GGM was plotted with the qgraph package^51^. We utilized the model to examine the interplay between biological variables, illness beliefs, and operation related expectations and QoL indicators. We estimated a partial correlation network and used LASSO (Least Absolute Shrinkage and Selection Operator) regularization^52^ to eliminate unreliable edges based on the Extended Gaussian Bayesian Information Criterion (EBIC-G)^53^. Therefore, we penalized overly complex models with a respective hyperparameter of γ = 0.5.

To estimate edge centrality, we calculated one-step expected influence (EI), a measure shown to better reflect strength centrality in psychological networks compared to traditional indicators^54^. EI is defined as the summed weight of a given node’s edges shared with the directly connected nodes in the network^54^.

We tested the stability of edge weights by creating 5000 samples with the bootnet package^44^using non-parametric bootstrapping (resampling rows with replacement). Case-drop bootstrapping (leaving a predefined proportion of the sample out) with 5000 samples was then used to test how much percent of our original sample could be dropped to maintain an average correlation of *r* > .7 of the node centrality estimates between the original and bootstrapped samples (“CS-coefficient”, minimum ≥ 0.25, recommended: ≥ 0.5)^44^.

Finally, we predefined “perceived current burden”, “expected postsurgical burden”, and “objective biological factors” as communities to enable a bridge analysis using bridge Expected Influence. Again, the networktools package^46^ was utilized. The concept of bridge EI is defined in a manner analogous to that of EI. However, in this case, the analysis is limited to edges that connect nodes within one (predefined) community to nodes within a different community.

### Directed acyclic graphs (DAGs)

In accordance with previous psychological research^55^, we estimated the Directed Acyclic Graphs (DAGs) using a modified hill-climbing algorithm^56,57^ implemented in the R package bnlearn^58^. This score-based algorithm draws bootstrapped samples to optimize a goodness-of-fit index by iteratively adding, removing, and reversing the edges of the network structure. Like in the GGM, we aimed to optimize the EBIC-G. To avoid local optimization issues, we restarted the process 50 times using 100 perturbations per restart (attempts to randomly insert/remove/reverse an edge)53). Afterwards, we drew 10,000 non-parametric bootstrapped samples (with replacement), estimated a network for each, and finally averaged the resulting networks to generate a final network structure. The final averaged network was achieved by first determining the strength of its edges and how often a directed edge (arrow) appeared with which orientation in the bootstrapped networks.

Only if an edge had a strength of greater than 0.85 and the final orientation was present in more than 50% (directional probability) of the bootstrapped networks^59^ it was used for the final DAGs. And second, as we favored edges with high specificity and assumed a rather sparse network structure, we applied a filter to networks that retains an arrow only if it is present in more than 85% of bootstrapped networks according to Sachs et al.^59^.

## Results

Descriptive statistics of the variables (before nonparanormal transformation), including mean (M), standard deviation (SD) are displayed in Table 1. A correlation matrix (S2), and a Gaussian Graphical Model of raw data before nonparanormal transformation and item indexing (S3) can be found in the supplementary materials.

**Table 1:** Sample baseline characteristics of the study population, overall and split up by type of surgery (CABG vs. valvular surgery).

		Total (*n* = 204)				Surgery type						
						CABG (*n* = 115)		Valvular (*n* = 89)				
Categorical Variables		n		%		n	%	n	%	χ2	df	p
Sex (female)		46		22.5		17	14.8	29	32.6	9.104	2	**0.003**
Education (high school)		33		16.2		27	23.5	6	6.7	9.17	1	**0.003**

Continuous Variables		M		SD		M	SD	M	SD	F	df	p
Age		64.01		9.69		65.86	8.27	61.62	10.85	10.051	1	**0.002**
Disability (PDI), MD = 3		22.1		14.54		23.56	15.54	20.21	15.69	2.489	1	0.116
Mental Quality of life (SF-12), MD = 7		48.35		10.99		48.97	10.59	47.55	11.49	0.836	1	0.362
Physical Quality of life (SF-12), MD = 5		39.08		10.37		38.87	10.14	39.34	10.72	0.104	1	0.747
Consequences (BIPQ 1), MD = 2		5.38		3.07		5.13	3.09	5.70	3.04	1.702	1	0.193
Timeline (BIPQ 2), MD = 7		3.44		2.97		2.89	2.84	3.44	2.97	9.270	1	**0.003**
Personal Control (BIPQ 3), MD = 3		4.18		2.66		4.29	2.84	4.04	2.42	0.426	1	0.515
Treatment Control (BIPQ 4), MD = 5		9.07		1.20		8.93	1.31	9.27	1.02	4.189	1	**0.042**
Identity (BIPQ 5), MD = 1		4.83		2.84		4.66	2.74	5.05	2.97	0.950	1	0.331
Concern (BIPQ 6), MD = 1		6.22		3.07		6.08	3.30	6.40	2.73	0.539	1	0.464
Coherence (BIPQ 7), MD = 3		7.33		2.65		7.02	2.83	7.74	2.34	3.752	1	0.054
Emotional response (BIPQ 8), MD = 1		4.83		3.02		4.51	3.03	5.24	2.97	2.920	1	0.089
Expected Disability (PDI-E), MD = 12		10.45		11.28		11.13	9.58	9.58	13.13	0.941	1	0.333
Expected Consequences (IPQ-E), MD = 11		9.36		3.16		9.71	2.78	8.90	3.55	3.317	1	0.070
Expected Personal Control (IPQ-E), MD = 11		14.52		2.69		14.80	2.34	14.17	3.06	2.795	1	0.096
Expected Treatment Control (IPQ-E), MD = 11		12.32		1.59		12.35	1.49	12.28	1.73	0.091	1	0.763
EuroSCORE II, MD = 11		1.61		1.38		1.43	0.8	1.83	1.86	4.314	1	**0.039**
BMI, MD = 3		29.01		5.64		29.38	5.51	28.54	5.82	1.119	1	0.291
CRP µg/ml, MD = 37		6.06		14.51		5.33	8.96	7.0	19.5	0.660	1	0.418

Significant values are in bold.

MD = Missing Data before imputation; All scores displayed are after imputation; Education = high school (vs. no high school); PDI = a modified version of the Pain Disability Index (range: 0 (minimum of observed values) − 59 (maximum of observed values)); SF-12 = Short Form Health Survey 12 (range: Mental SF-12: 18.5–67.2; Physical SF-12: 14.2–63.3); BIPQ = Brief Illness Perception Questionnaire (range: for the subscales Consequences Timeline, Personal Control, Identity, Concern, Coherence, Emotional response: 0–10, for the subscale Treatment Control: 5–10); PDI-E = Expected version of the modified Pain Disability Index (range: 0–48); IPQ-E = Expected Illness Perception Questionnaire (subscale Personal Control: 4–20, subscale Treatment Control: 8–15, subscale Consequences: 4–20); EuroSCORE = European System for Cardiac Operative Risk Evaluation (range: 0.5–11.5); BMI = Body Mass Index (range: 17.1–59.3); CRP = C-reactive protein (0.06–146 µg/ml).

Patients scheduled for coronary artery bypass grafting (CABG) and those scheduled for valvular surgery exhibited significant differences regarding sex, age, their illness beliefs concerning the timeline and treatment control, as well as in the EuroSCORE II. The CABG sample indicated a lower percentage of women, was older, while CABG patients perceived their cardiac illness as having a shorter duration and believed that the surgery would provide slightly less benefits and had a lower EuroSCORE II compared to patients undergoing valvular surgery. All other variables did not differ significantly between samples.

### Gaussian graphical model (GGM)

#### Topology and notable connections

**Fig. 1:**
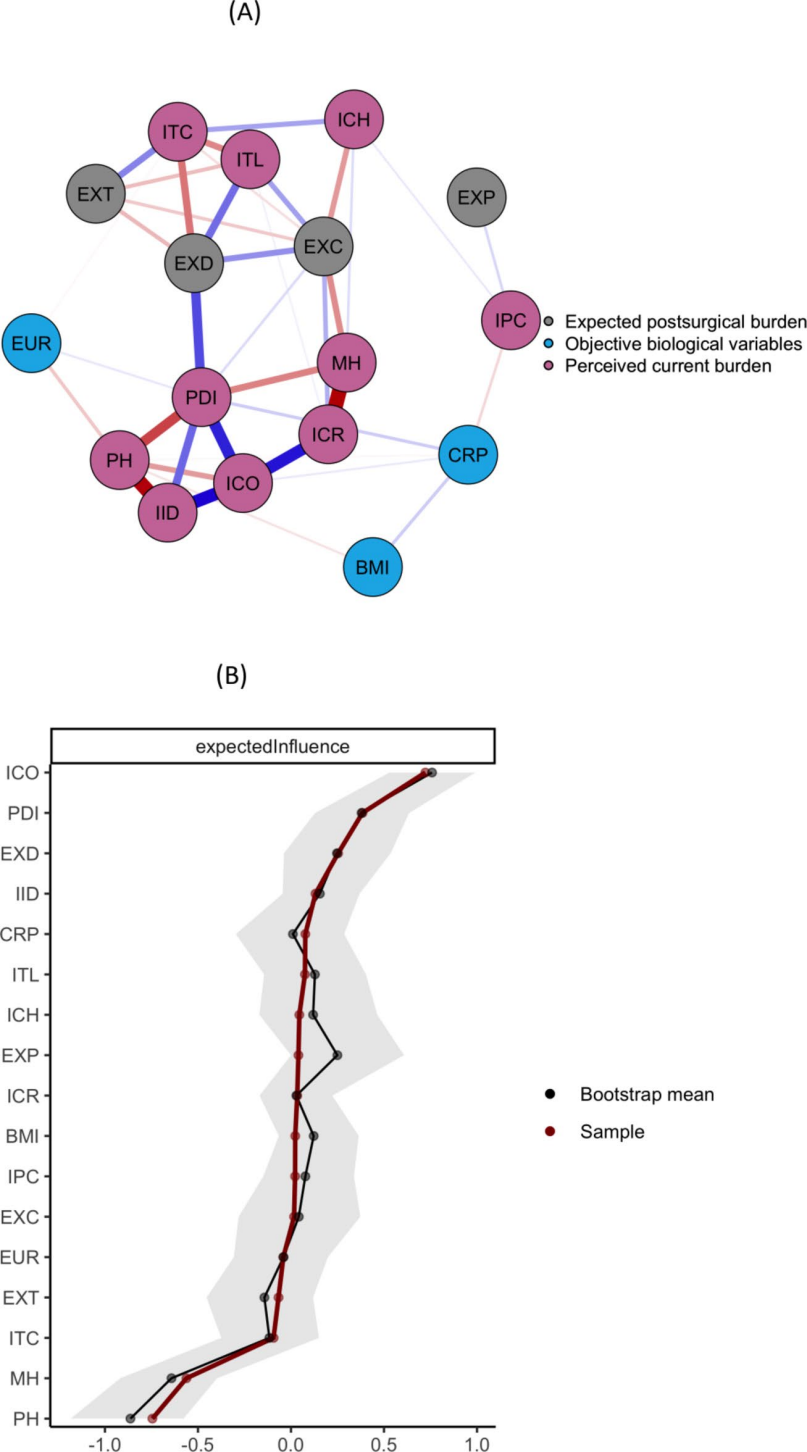
(**A**) Gaussian Graphical Model of Biopsychological Variables in Patients undergoing Cardiac Surgery. (**B**) Expected Influence of Biopsychological Variables in Patients undergoing Cardiac Surgery. BMI = Body Mass Index, CRP = C-reactive protein, EUR = EuroSCORE II - Risk of 30-day mortality, EXC = Illness Perception Expectations: Consequences, EXD = Pain Disability Index: Expectations (sum), EXP = Illness Perception Expectations: Personal Control, EXT = Illness Perception Expectations: Treatment Control, ICH = Illness Perception: Coherence (Item 7 BIPQ), ICO = Illness Perception: Consequences (Item 1 BIPQ), ICR = Illness Perception: Concern & Emotional Response (Item 6 + 8, BIPQ), IID = Illness Perception: Identity (Item 5 BIPQ), IPC = Illness Perception: Personal Control (Item 3 BIPQ), ITC = Illness Perception: Treatment Control (Item 4 BIPQ), ITL = Illness Perception: Timeline (Item 2 BIPQ), MH = Short Form Health: Current Mental Quality of Life (sum), PDI = Pain Disability Index: Current Disability (sum), PH = Short Form Health: Current Physical Quality of Life (sum). (**A**) Red edges depict negative partial correlations. Blue edges depict positive partial correlations. (**B**) Nodes are sorted by one-step Expected Influence. Shaded areas indicate 95% bootstrapped confidence interval (CI). A large and positive Expected Influence implies strong and positively signed connections. A large and negative Expected Influence implies strong and negatively signed connections. Expected Influence estimations near zero or a CI overlapping zero imply weak and possibly spurious connections.

Figure 1A displays the regularized Gaussian graphical model (GGM) cross-sectional network. The overall network connectivity was rather sparse (38 of 136 possible edges, 27.9%). In general, the model indicated strong associations between different psychological variables. In contrast, the medical variables (BMI, CRP, EUR) did not show strong associations with psychological aspects, at least not in this sample.

In detail, patients’ illness perception of consequences (ICO) showed strong positive associations with current illness-related disability (PDI), the perception of symptom attribution to the cardiac condition (IID), and concern and emotional response to the illness (ICR). The illness perception identity (IID) was strongly and negatively associated with patients’ physical health (PH).

Illness-related disability, assessed by the PDI sum score, exhibited strong negative associations with physical health (PH) and weaker negative associations with mental health (MH). Disability also showed a strong positive association with expected disability (EXD) six months post-surgery and a weaker association with illness perception identity (IID).

Patients’ concern and emotional response to their illness (ICR) indicated a strong negative association with mental health and appeared to link perceived consequences of the cardiac condition (ICO) with mental quality of life (MH). Additionally, mental health (MH) showed a negative association with expected consequences of cardiac disease six months post-surgery (EXC).

Expected disability at six months follow-up after surgery was positively associated with expected consequences of the cardiac disease (EXC) and disease timeline (ITL), and negatively associated with the perception of (current) treatment control (ITC) and expected positive impact of surgery (EXT).

The perception of coherence (ICH), reflecting patients’ understanding of their disease, indicated a positive association with treatment control (ITC) and a negative association with expected consequences (EXC) six months post-surgery. Interestingly, both nodes related to personal control exhibited only a few weak associations with other variables in the network.

### Node importance

Figure 1B shows the individuals’ nodes importance due to the node’s Expected Influence: Analyses indicated that the strongest positive EI exerted the perceived consequences of the illness (ICO), followed by the current illness-related disability (PDI), and the expected illness-related disability (EXD). The strongest negative EI exerted physical and mental health (PH and MH). The EI results were comparably stable according to the CS-coefficient (0.51, see also Figure S4). For the exact partial correlations, refer to Table T1 (supplementary material).

### Communities and bridges

**Fig. 2:**
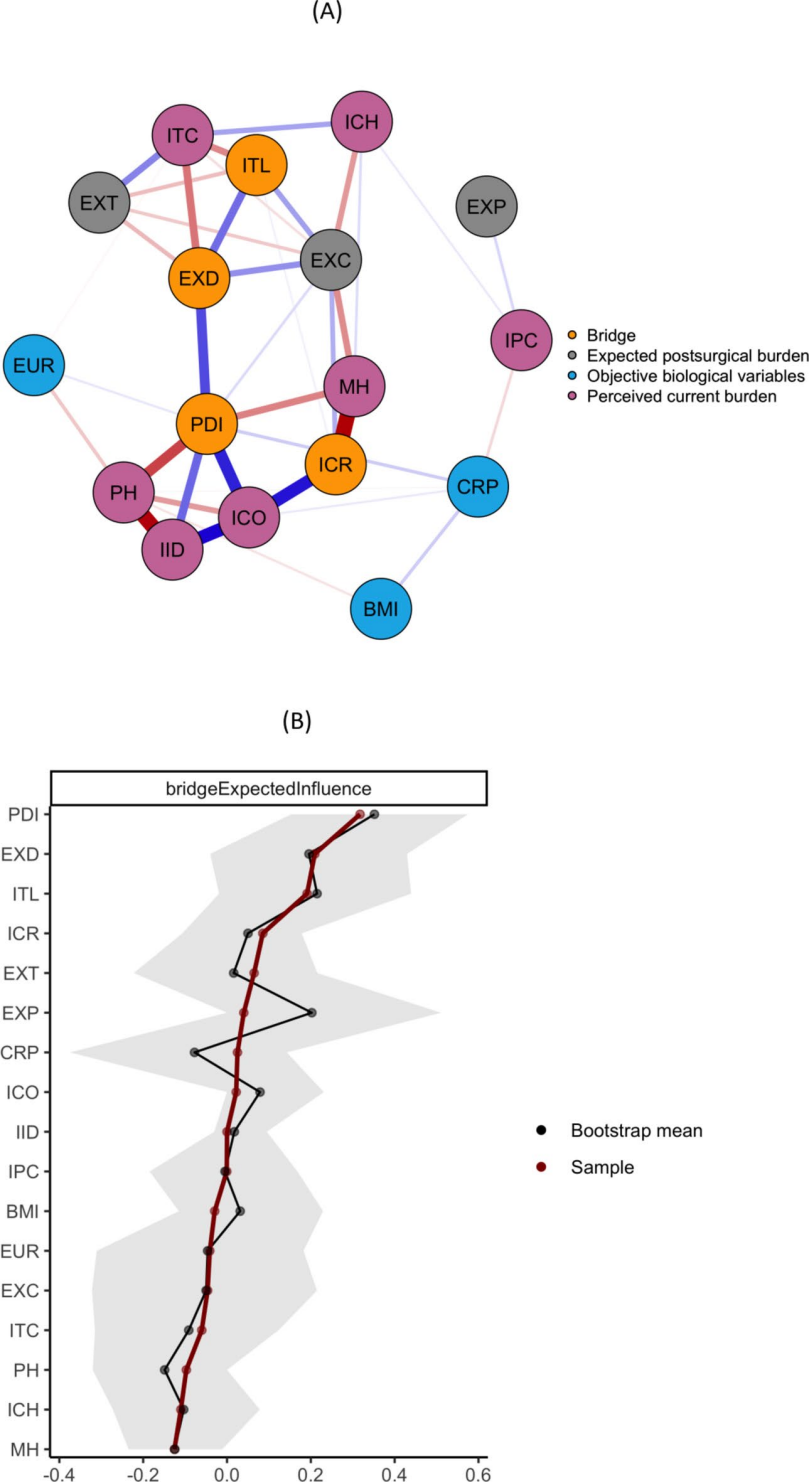
(**A**) Gaussian Graphical Model with Highlighted Bridge Nodes of Biopsychological Variables in Patients undergoing Cardiac Surgery. (**B**) Bridge Expected Influence of Biopsychological Variables in Patients undergoing Cardiac Surgery. BMI = Body Mass Index, CRP = C-reactive protein, EUR = EuroSCORE II - Risk of 30-day mortality, EXC = Illness Perception Expectations: Consequences, EXD = Pain Disability Index: Expectations (sum), EXP = Illness Perception Expectations: Personal Control, EXT = Illness Perception Expectations: Treatment Control, ICH = Illness Perception: Coherence (Item 7 BIPQ), ICO = Illness Perception: Consequences (Item 1 BIPQ), ICR = Illness Perception: Concern & Emotional Response (Item 6 + 8, BIPQ), IID = Illness Perception: Identity (Item 5 BIPQ), IPC = Illness Perception: Personal Control (Item 3 BIPQ), ITC = Illness Perception: Treatment Control (Item 4 BIPQ), ITL = Illness Perception: Timeline (Item 2 BIPQ), MH = Short Form Health: Current Mental Quality of Life (sum), PDI = Pain Disability Index: Current Disability (sum), PH = Short Form Health: Current Physical Quality of Life (sum). (**A**) Red edges depict negative partial correlations. Blue edges depict positive partial correlations. (**B**) Nodes are sorted by one-step Bridge Expected Influence. Shaded areas indicate 95% bootstrapped confidence interval (CI). A large and positive Expected Influence implies strong and positively signed connections. A large and negative Expected Influence implies strong and negatively signed connections. Expected Influence estimations near zero or a CI overlapping zero imply weak and possibly spurious connections.

Detected bridge-nodes according to our prespecified communities, as well as unstandardized estimates of one-step Bridge Expected Influence can be found in Fig. 2A and B. The detected bridge nodes indicating the connection between different systems are the current illness-related disability (PDI), the expected illness-related disability for the time-point 6 months after surgery (EXD), the perception of the disease’s timeline (ITL), and patients’ concern and emotional response (ICR). In particular, the illness-related disability (PDI) and the expected disability (EXD) represent a bridge with the highest bridge EI and a positive correlation. They mainly connect the “perceived current burden” community with the “expected postsurgical burden” community. The stability of the bridge EI was mediocre (CS-coefficient: 0.28; see also Figure S5), but still high enough to enable interpretability (≥ 0.25).

### Directed acyclic graphs (DAGs)

**Fig. 3:**
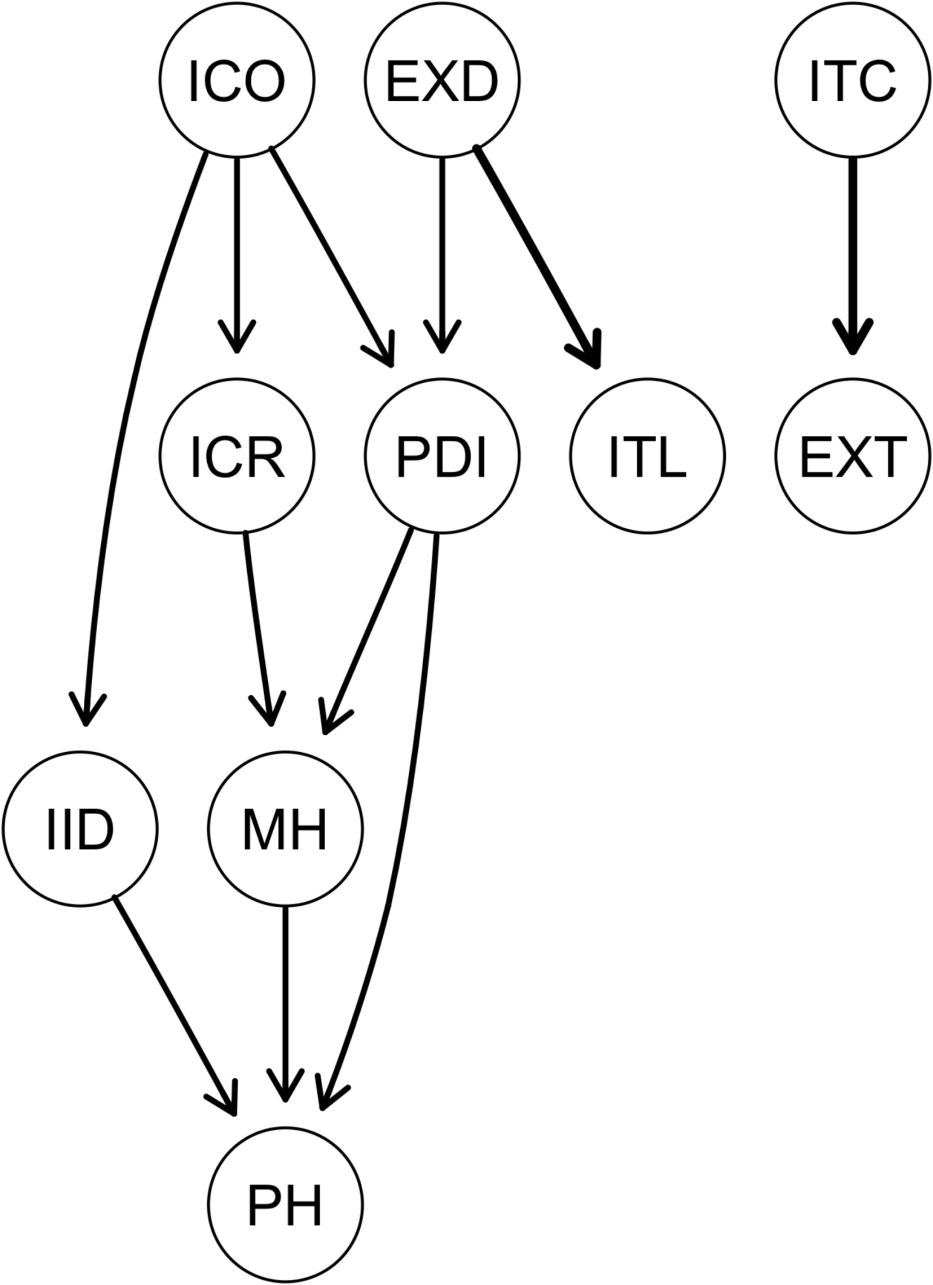
Directed Acyclic Graph with Arrows Depicting Directional Probability of Biopsychological Variables in Patients undergoing Cardiac Surgery. BMI = Body Mass Index, CRP = C-reactive protein, EUR = EuroSCORE II - Risk of 30-day mortality, EXC = Illness Perception Expectations: Consequences, EXD = Pain Disability Index: Expectations (sum), EXP = Illness Perception Expectations: Personal Control, EXT = Illness Perception Expectations: Treatment Control, ICH = Illness Perception: Coherence (Item 7 BIPQ), ICO = Illness Perception: Consequences (Item 1 BIPQ), ICR = Illness Perception: Concern & Emotional Response (Item 6 + 8, BIPQ), IID = Illness Perception: Identity (Item 5 BIPQ), IPC = Illness Perception: Personal Control (Item 3 BIPQ), ITC = Illness Perception: Treatment Control (Item 4 BIPQ), ITL = Illness Perception: Timeline (Item 2 BIPQ), MH = Short Form Health: Current Mental Quality of Life (sum), PDI = Pain Disability Index: Current Disability (sum), PH = Short Form Health: Current Physical Quality of Life (sum). The nodes BMI, CRP, EUR, EXC, EXP, ICH, IPC are not depicted here as they were not connected to any other node. The thickness of the edges indicates the directional probability - the proportion of averaged bootstrapped networks where this edge points in this direction yielding an estimate of confidence in the directionality of the connection.

The final DAGs using a directed probability threshold of 0.85^59^ are shown in Fig. 3 (Bayesian Information Criterion (BIC) and Directional Probabilities Values of the Arrows in the Directed Acyclic Graph (DAG) are displayed in T2, supplementary material). The thickness of the edges in Fig. 3 indicates the directional probability (dp) - the proportion of averaged bootstrapped networks where this edge points in this direction yielding an estimate of confidence in the directionality of the connection. The three most important edges according to the directional probability are the connection between the sum score of the Pain Disability Index - Expectations (EXD) and the perceived illness timeline (ITL; dp = 0.73), the connection between the perceived treatment control (ITC) and the expected treatment control (EXT; dp = 0.68), and the connection between the concern & emotional response indexed item (ICR) and mental health (MH; dp = 0.66).

The directional graph (Fig. 3) also reveals an important upstream node (in the sense of a “common cause”) of the perception of illness consequences (ICO) influencing the diseases’ identity (meaning the amount symptoms attributed to the disease; IID, BIC = -54.57, dp = 0.53), the illness-related disability (PDI, BIC = -51.54, dp = 0.65), and the patients’ concern and emotional response to the illness (ICR, BIC = -30.33, dp = 0.65). Importantly, patients’ concern and emotional response to the illness (ICR, BIC = -22.08, dp = 0.66) as well as patients’ perceived disability (PDI, BIC = -0.39, dp = 0.53) seem to influence patients mental quality of life (MH), while patients’ mental quality of life (MH, BIC = -8.9, dp = 0.53) and the perceived identity of the disease (IID, BIC = -19.46, dp = 0.6) influence physical functioning (PH). Expected disability (EXD) is the starting point to a chain of nodes with downstream associations with current disability (PDI, BIC = -16.78, dp = 0.53) and perceived illness timeline (ITL, BIC = -10.68, dp = 0.73). Current treatment control (ITC) has a downstream association with expected treatment control (EXT, BIC = -5.91, dp = 0.68). Associations of objective indicators of physical impairment such as the BMI, EUR, and CRP with perceived current or expected postsurgical impairment are absent. Also, expected consequences (EXC), EXP (expected personal control), coherence (ICH), and personal control (IPC) are not connected in the DAG.

## Discussion

Our study is the first to examine the network structure of both psychological variables (QoL assessments, perceived illness-related disability, and illness perceptions and expectations) and biomedical variables in patients before undergoing cardiac surgery. We utilized a Gaussian Graphical Model (GGM) and a Directed Acyclic Graph (DAG) to explore these interactions. In general, our findings reveal that patients’ mental and physical QoL are strongly associated with psychological variables but appear only weakly associated to biomedical variables such as risk scores (EuroSCORE II) and inflammatory markers (i.e., CRP).

A key finding was the strong influence of current disability, or the consequences experienced due to the cardiac condition, on the network structure. In the GGM, it exhibited the greatest EI, while in the DAG, it was an important parent node to several other nodes. Taken together, these findings indicate that current illness-related disability represents an interesting proxy for general illness-related symptoms, thereby underscoring the necessity for its routine assessment in future studies.

Furthermore, both current and expected illness related disability demonstrated high bridge centrality estimates. Also, both nodes exhibit a substantial unique association with each other. Converging evidence from the DAG suggested that disability expectations act as an influential parent node, likely affecting current disability in this group of patients. The DAG further indicated that patients’ expected disability and current perceived disability influence their mental and physical QoL more significantly than vice versa. This finding points in the direction that optimizing preoperative treatment and disability expectations may have a positive impact on patients’ QoL.

In summary, our findings underscore the relevance of expectations and illness perceptions, even in cardiac surgery patients, and suggest that optimizing patients’ expectations may foster beneficial postsurgical outcomes.

The GGM indicated robust associations among different psychological variables: Specifically, the illness-related disability (PDI) showed strong positive associations with the expected disability six months after surgery (EXD), suggesting that patients who perceive greater disability from their illness also tend to *expect* higher levels of disability or vice versa. Conversely, current disability was negatively associated with patients’ physical QoL (PH) and to a lesser extent with mental QoL (MH). Interestingly, objective biological indicators of symptom severity (EUR, BMI, CRP) neither showed strong, nor consistent associations with these expectations or perceived disability in the GGM and DAG. The finding that both QoL subscales were strongly associated with psychological variables, but not with biomedical variables, underscores the importance of considering patients’ illness perceptions, subjective disability assessments, and expectations in the context of invasive medical treatments. This finding is consistent with other work suggesting that patients’ illness beliefs and expectations before cardiac surgery predict outcomes after cardiac surgery independent of objective medical risk factors^7,25,60^.

Perceived current disability in cardiac patients demonstrated consistent associations with expected postsurgical disability. Bridge EI indicated that both illness-related disability (PDI) and its expectation version (EXD) represent strong bridges connecting the perceived current burden community with the expected postsurgical burden community. This advocates for the validity of the PDI and the expected PDI and suggests that illness expectations are strongly associated with the current subjective illness burden. In the DAG, identifying the perception of illness consequences (ICO) as a key upstream node underscores its relevant influence on patients’ illness identity, disability, and emotional response. The expected disability (EXD) also served as a starting point in a chain of downstream associations affecting current disability (PDI) and perceived illness timeline (ITL). This sequential relationship suggests that an early management of expectations may lead to positive effects on other aspects of patients’ health perceptions and outcomes. Similarly, the current treatment control (ITC) shows downstream associations with expected treatment control (EXT), indicating that improving current treatment experiences could enhance future treatment expectations. There is promising (preliminary) evidence that optimizing preoperative expectations may be capable of optimizing outcomes after cardiac surgery. For instance, in the PSY-HEART I trial, a brief preoperative psychological intervention aiming to optimize patients’ preoperative expectations led to improved disability, mental quality of life and led to less inflammation compared to standard medical care only in patients undergoing coronary artery bypass graft surgery^28^.

Several variables, including expected consequences (EXC), expected personal control (EXP), current personal control (IPC), and coherence (ICH), did not show reliable connections in the DAG. The lack of influence of patients’ personal control is particularly noteworthy, as personal control is often considered a key variable in health-related behaviors (e.g., physical activity) and for QoL^61^. One possible explanation for this finding could be the timing of our assessment. Since we used baseline data, it may be functional for patients not to place too much emphasis on their own efforts shortly before cardiac surgery, given their passive role during the surgery itself. In contrast, personal control may become crucial after surgery, as engaging in health-related behaviors is vital for optimal recovery.

Instead of focusing on personal control expectations before surgery, our results advocate for improving patients’ perceived and expected treatment control. The GGM indicated substantial associations between treatment control and expected disability, which in turn, as discussed above, was shown to be a central node in the patients’ network and seemed to influence current disability and QoL variables. This finding is in line with findings from placebo research indicating the prominent role of treatment (outcome) expectations as one crucial mechanism driving placebo effects^19,62^.

Clinical implications.

Our findings encourage that targeting patients’ baseline expectations may be beneficial for improving postoperative QoL. However, it remains largely unknown which specific expectations should be targeted. Given the variety of different expectations^22^, this poses a challenge, though this paper provides some valuable insights. Integrating psychological considerations into cardiac surgery patients’ preparation may be relevant for clinical practice. While guidelines such as Early Recovery After Surgery (ERAS) acknowledge the importance of aspects such as structured preoperative information and patient engagement to reduce anxiety and increase compliance with the protocol^63^, those are often not optimally addressed due to time constraints. Incorporating mental health professionals into the cardiosurgery unit may further improve patient care.

## Limitations

Generally, this study is a post-hoc re-analysis of existing cross-sectional data limiting the quality of evidence. For instance, some relevant variables such as depressive symptoms were absent. Other relevant variables, such as education and gender, were not included in the analyses as potentially interesting moderators due to limitations in sample size. Additionally, since this study focused exclusively on biopsychological variables, the social aspect is missing, which limits the ability to fully understand the holistic picture provided by a biopsychosocial approach.Future studies could take such an approach to find out whether our results hold in a larger sample and with a higher variety of included variables. Another limitation of this study is that we did not control for differences in the timing of preoperative assessments, which varied between approximately seven days before surgery in the PSY-HEART I trial and one day prior to surgery in the ValvEx study. This variability may have influenced patients’ responses and should be considered when interpreting the results.

While this study focused on cardiac surgery patients, the psychological variables examined, such as illness perceptions and expectations, are likely relevant across various medical and surgical contexts. Prior research has shown that these factors significantly influence quality of life and recovery outcomes in diverse patient groups, suggesting some degree of generalizability. However, the medical variables included in our analysis, such as EuroSCORE II and C-reactive protein (CRP), are specific to cardiac surgery and may not be as applicable to non-cardiac populations.

Moreover, the interplay between psychological and medical factors may differ depending on the type and severity of the surgical procedure, as well as demographic and clinical characteristics of the population. Future research should extend this network-based approach to other surgical or non-surgical patient groups, incorporating condition-specific medical factors while retaining psychological variables, to explore the broader applicability of biopsychological determinants of quality of life.

There are some caveats to consider when using network models in this context: First, we used imputation strategies which have not yet been studied in detail for network models^64^. This may have impaired the validity especially of the smaller edges in the network. To account for this shortcoming, our discussion focuses on the larger, more robust edges. Second, we used single items alongside sum-scores to grasp the relevant concepts. This may have introduced some topological overlap, as rather broad sum scores were related to narrower single item questions. However, we did use appropriate techniques to check for overlap between variables. Also, the single item nodes were not continuous, but rather ordinal. As is the case with numerous other network analyses, they were presumed to be continuous for the estimations, given the absence of established ordinal models. Third, the network models included variables from different levels of analysis (medical/biological, psychological, mixed). This may pose a problem to the results’ generalizability as the data do not stem from a circumscribed closed system where “emergent topological properties”^65^ could be ruled out. A more specific limitation of DAG network models is that they can, by design, only focus on acyclic connections but the real causal structure probably also includes vicious circles that cannot be adequately modeled. Therefore future studies could use panel networks^23^ and/or intensive longitudinal modeling^66^. Lastly, a formal theory for this complex system is missing^67^. Hence, the results are exploratory in nature.

## Conclusion

This network analysis highlights the crucial role of illness perceptions and the current and expected perceived disability (independent from biomedical variables) for preoperative QoL as one of the most important predictors of postoperative QoL in patients undergoing cardiac surgery. Interventions aimed at modifying illness perceptions and managing expectations could potentially lead to enhanced postsurgical physical and mental health outcomes and reduced postsurgical perceived disability. A biopsychosocial model should be considered in prehabilitation and cardiac surgery to provide patients with the best healthcare possible.

## Electronic supplementary material

Below is the link to the electronic supplementary material.

Supplementary Material 1

Supplementary Material 2

Supplementary Material 3

Supplementary Material 4

Supplementary Material 5

Supplementary Material 6

Supplementary Material 7

Supplementary Material 8

Supplementary Material 9

## Data Availability

Data can be requested by the corresponding author.

## Declarations

### Study approval

Ethical approval for both studies (PSY-HEART I trial: AZ 150/10, ValvEx trial: AZ rastan-4620) was obtained from the institutional review board of the Medical School, University of Marburg.
