# Supplementary figures and images for: A biopsychological network approach to variables contributing to preoperative quality of life in patients undergoing cardiac surgery
Source: Sci Rep. 2025 Mar 13;15:8746. doi: 10.1038/s41598-025-93467-7 (PMC11906646; doi:10.1038/s41598-025-93467-7)

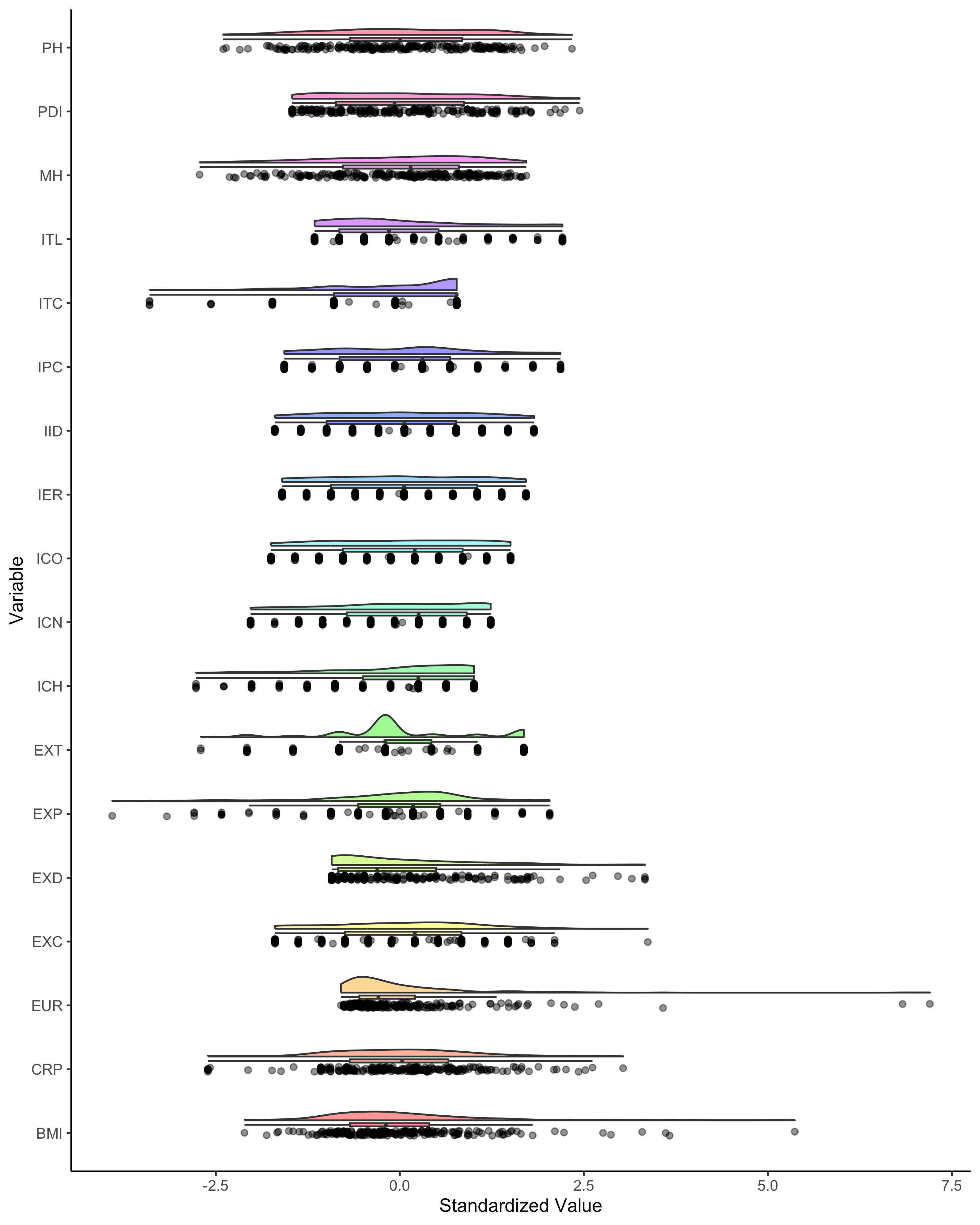

Supplement: Supplementary file 1 — Supplementary Material 1 [file 41598_2025_93467_MOESM1_ESM.jpg]

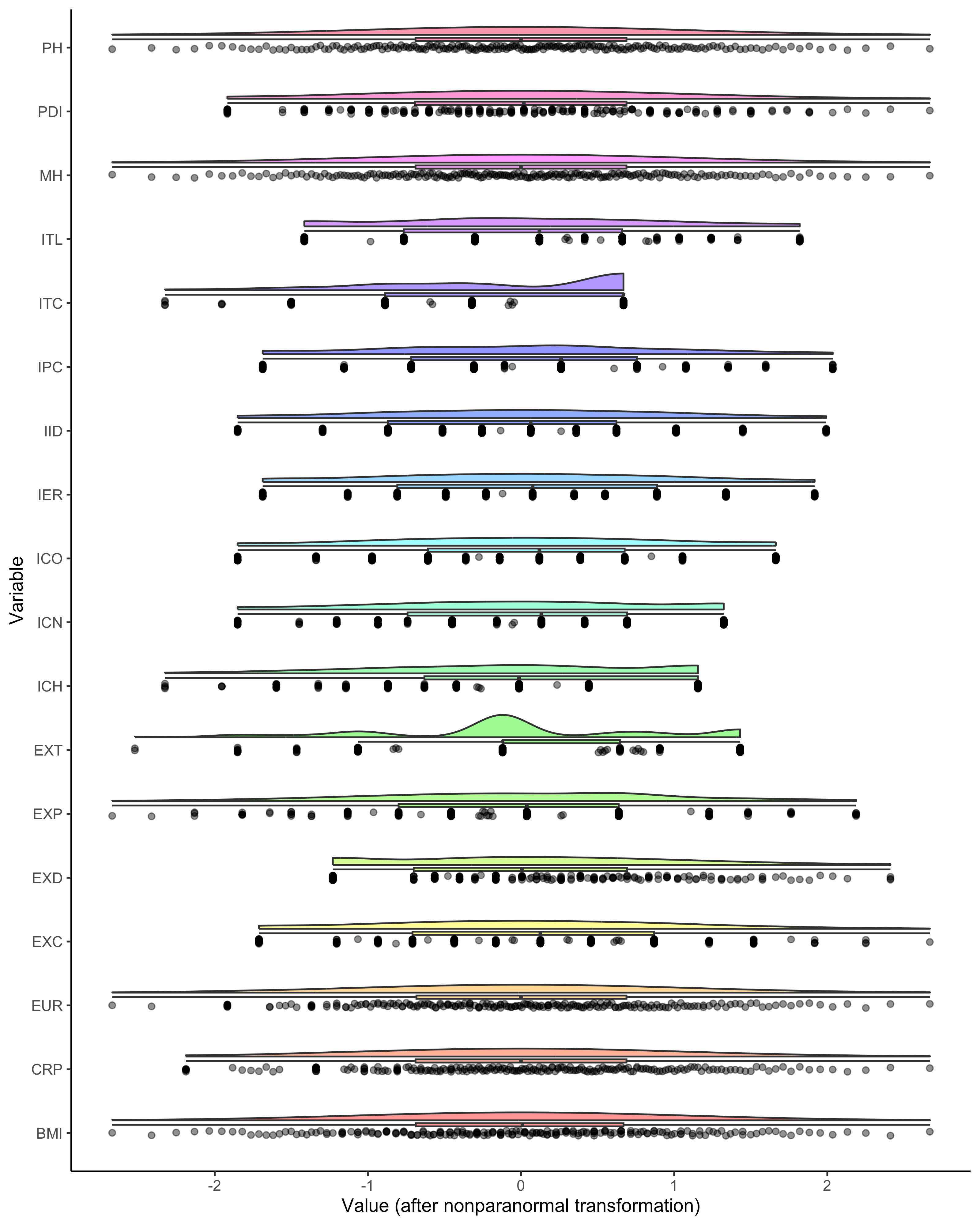

Supplement: Supplementary file 2 — Supplementary Material 2 [file 41598_2025_93467_MOESM2_ESM.jpg]

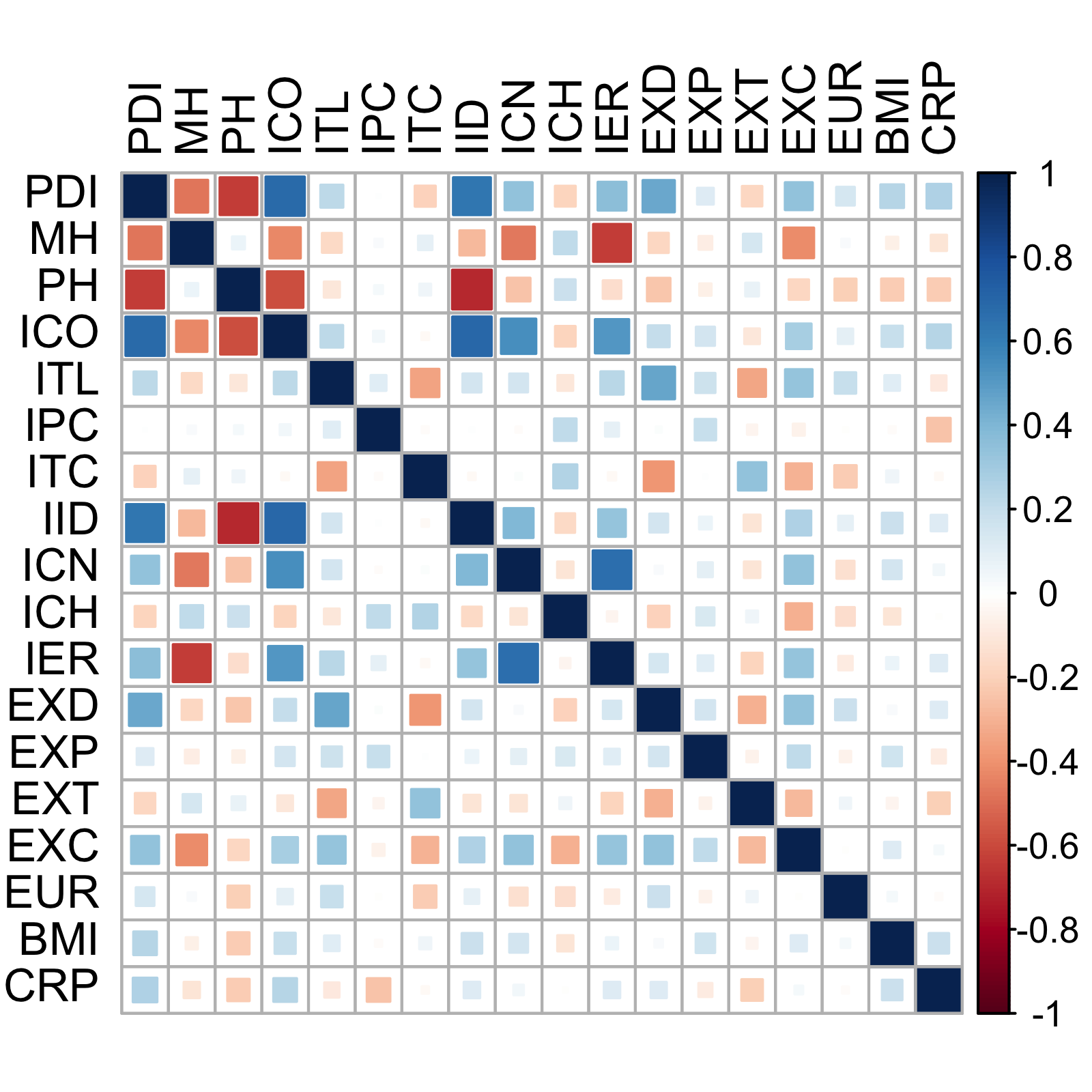

Supplement: Supplementary file 3 — Supplementary Material 3 [file 41598_2025_93467_MOESM3_ESM.tiff]

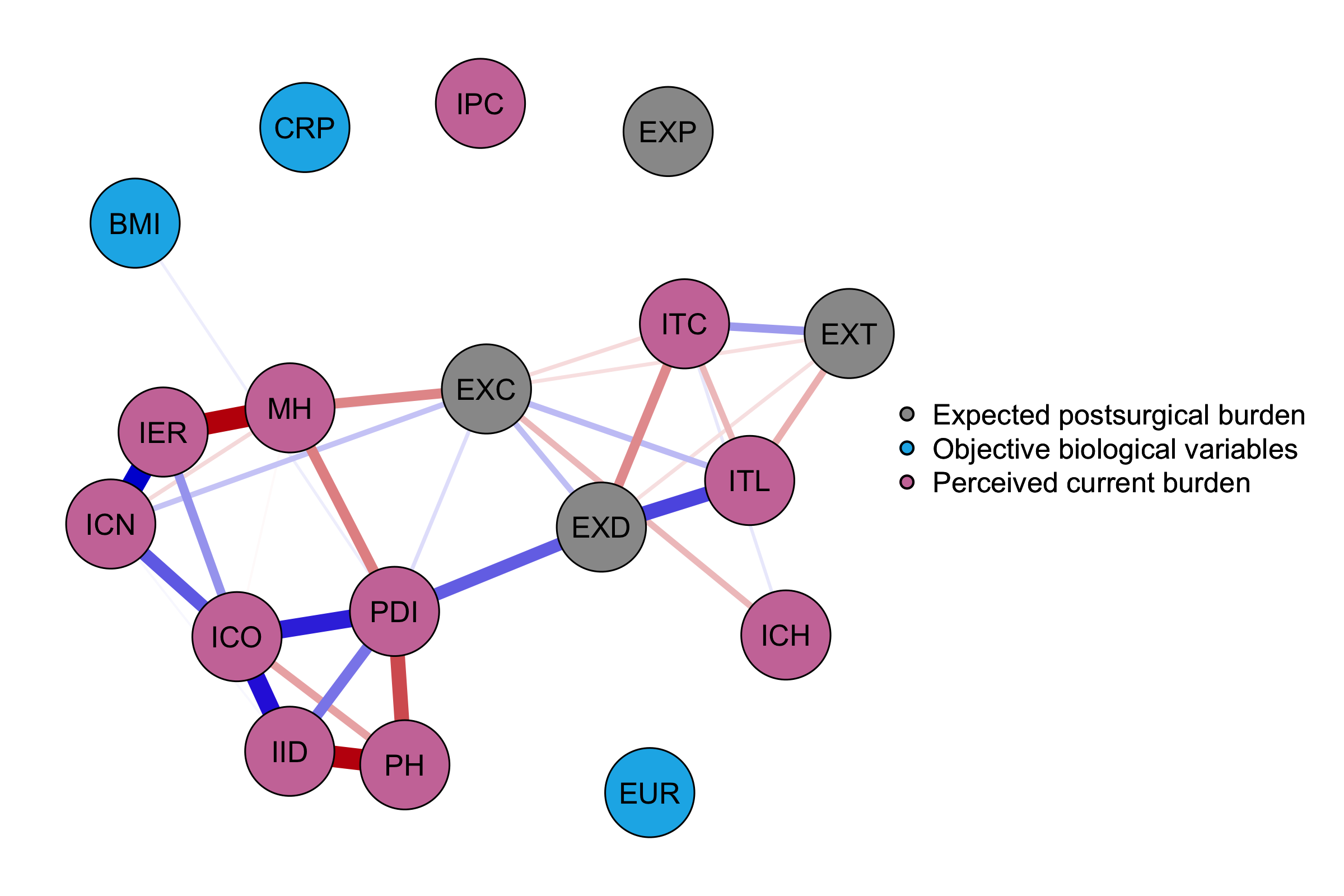

Supplement: Supplementary file 4 — Supplementary Material 4 [file 41598_2025_93467_MOESM4_ESM.tiff]

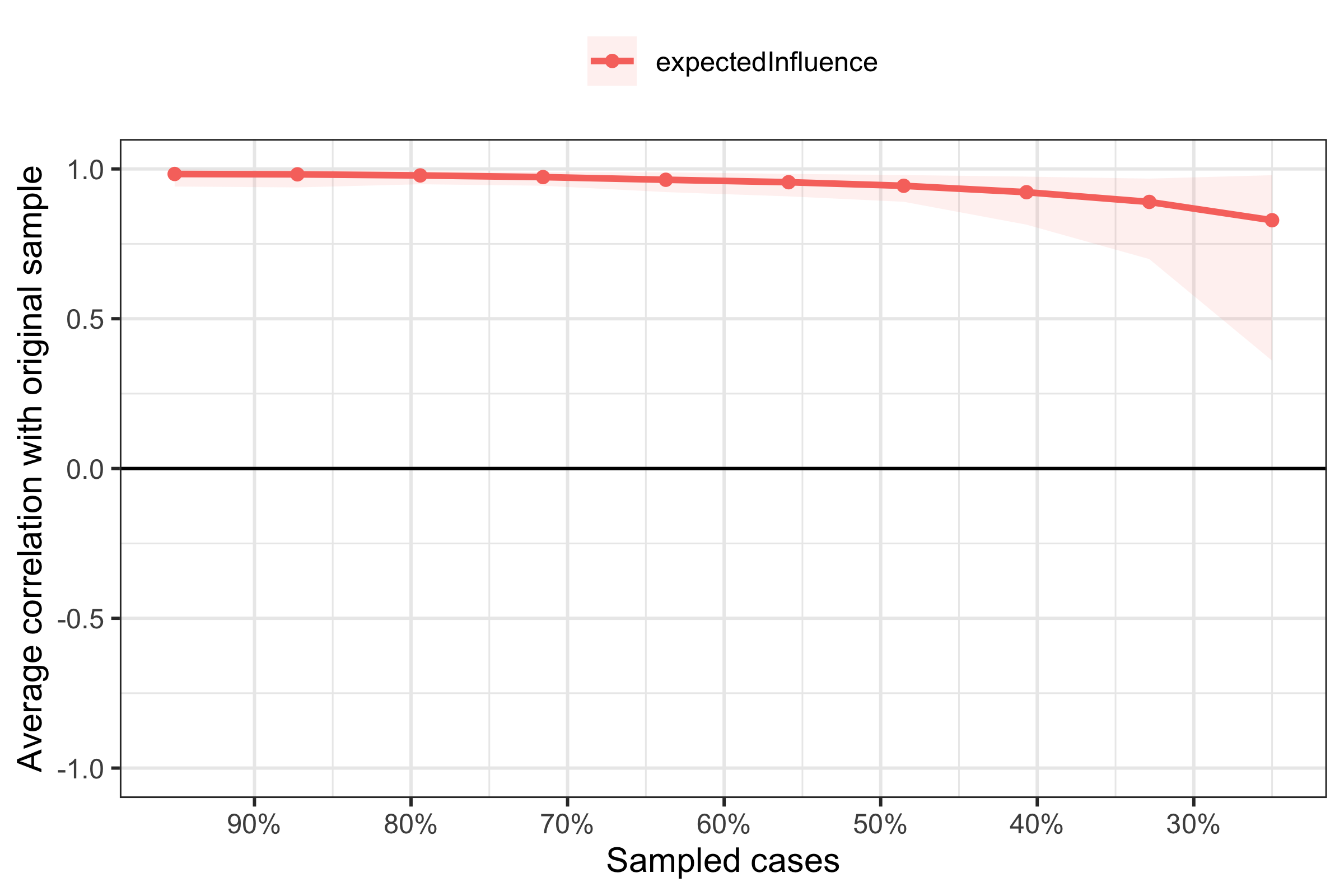

Supplement: Supplementary file 5 — Supplementary Material 5 [file 41598_2025_93467_MOESM5_ESM.tiff]

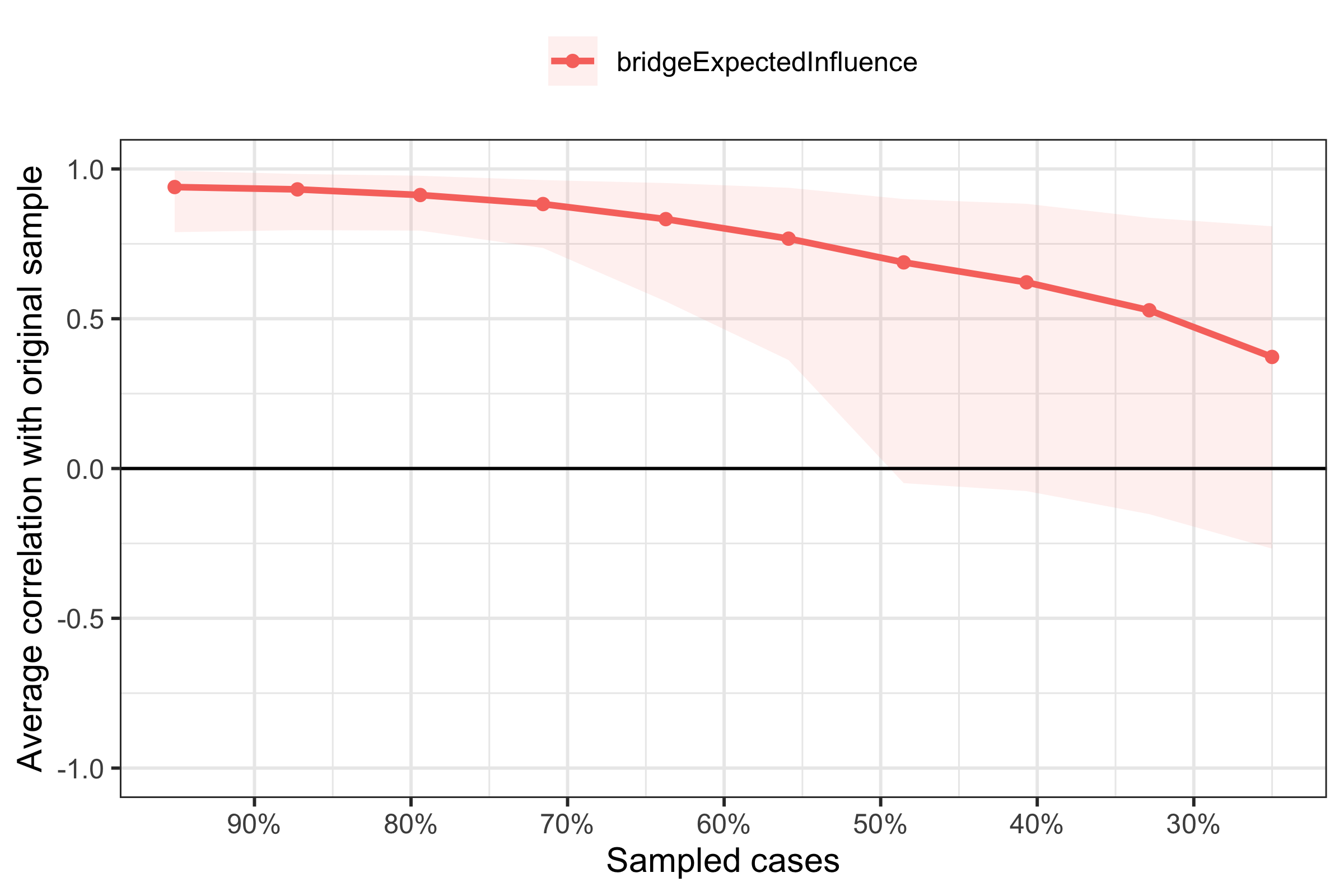

Supplement: Supplementary file 6 — Supplementary Material 6 [file 41598_2025_93467_MOESM6_ESM.tiff]
