# Supplementary material for: A biopsychological network approach to variables contributing to preoperative quality of life in patients undergoing cardiac surgery
Source: Sci Rep. 2025 Mar 13;15:8746. doi: 10.1038/s41598-025-93467-7 (PMC11906646; doi:10.1038/s41598-025-93467-7)
Supplement: Supplementary file 7 — Supplementary Material 7 [file 41598_2025_93467_MOESM7_ESM.docx]

**Table T1.** Weight matrix of Gaussian Graphical Model of raw data after nonparanormal transformation and item indexing

**PDI MH PH ICO ITL IPC ITC IID ICH ICR EXD EXP EXT EXC EUR BMI CRP**

**PDI** 0.00 -0.14 -0.22 0.25 0.00 0.00 0.00 0.17 0.00 0.00 0.20 0.00 0.00 0.04 0.03 0.00 0.05

**MH** -0.14 0.00 0.00 0.00 0.00 0.00 0.00 0.00 0.04 -0.33 0.00 0.00 0.00 -0.13 0.00 0.00 0.00

**PH** -0.22 0.00 0.00 -0.11 0.00 0.00 0.00 -0.32 0.00 0.00 0.00 0.00 0.00 0.00 -0.06 -0.03 -0.01

**ICO** 0.25 0.00 -0.11 0.00 0.00 0.00 0.00 0.29 0.00 0.27 0.00 0.00 0.00 0.00 0.00 0.00 0.02

**ITL** 0.00 0.00 0.00 0.00 0.00 0.00 -0.13 0.00 0.00 0.01 0.16 0.00 -0.07 0.10 0.00 0.00 0.00

**IPC** 0.00 0.00 0.00 0.00 0.00 0.00 0.00 0.00 0.02 0.00 0.00 0.04 0.00 0.00 0.00 0.00 -0.04

**ITC** 0.00 0.00 0.00 0.00 -0.13 0.00 0.00 0.00 0.10 0.00 -0.15 0.00 0.13 -0.03 -0.01 0.00 0.00

**IID** 0.17 0.00 -0.32 0.29 0.00 0.00 0.00 0.00 0.00 0.00 0.00 0.00 0.00 0.00 0.00 0.00 0.00

**ICH** 0.00 0.04 0.00 0.00 0.00 0.02 0.10 0.00 0.00 0.00 0.00 0.00 0.00 -0.11 0.00 0.00 0.00

**ICR** 0.00 -0.33 0.00 0.27 0.01 0.00 0.00 0.00 0.00 0.00 0.00 0.00 0.00 0.09 0.00 0.00 0.00

**EXD** 0.20 0.00 0.00 0.00 0.16 0.00 -0.15 0.00 0.00 0.00 0.00 0.00 -0.08 0.12 0.00 0.00 0.00

**EXP** 0.00 0.00 0.00 0.00 0.00 0.04 0.00 0.00 0.00 0.00 0.00 0.00 0.00 0.00 0.00 0.00 0.00

**EXT** 0.00 0.00 0.00 0.00 -0.07 0.00 0.13 0.00 0.00 0.00 -0.08 0.00 0.00 -0.06 0.00 0.00 0.00

**EXC** 0.04 -0.13 0.00 0.00 0.10 0.00 -0.03 0.00 -0.11 0.09 0.12 0.00 -0.06 0.00 0.00 0.00 0.00

**EUR** 0.03 0.00 -0.06 0.00 0.00 0.00 -0.01 0.00 0.00 0.00 0.00 0.00 0.00 0.00 0.00 0.00 0.00

**BMI** 0.00 0.00 -0.03 0.00 0.00 0.00 0.00 0.00 0.00 0.00 0.00 0.00 0.00 0.00 0.00 0.00 0.05

**CRP** 0.05 0.00 -0.01 0.02 0.00 -0.04 0.00 0.00 0.00 0.00 0.00 0.00 0.00 0.00 0.00 0.05 0.00

***Note.*** Cells contain standardized edge weights (partial correlations).
