# Supplementary material for: A biopsychological network approach to variables contributing to preoperative quality of life in patients undergoing cardiac surgery
Source: Sci Rep. 2025 Mar 13;15:8746. doi: 10.1038/s41598-025-93467-7 (PMC11906646; doi:10.1038/s41598-025-93467-7)
Supplement: Supplementary file 8 — Supplementary Material 8 [file 41598_2025_93467_MOESM8_ESM.docx]

**T2.** Bayesian Information Criterion (BIC) and Directional Probabilities Values of the Arrows in the Directed Acyclic Graph (DAG).

| From node | To node | BIC | Directional Probability |
| --- | --- | --- | --- |
| ICO | IID | -54.57 | 0.53 |
| ICO | PDI | -51.54 | 0.65 |
| ICO | ICR | -30.33 | 0.65 |
| ICR | MH | -22.08 | 0.66 |
| IID | PH | -19.46 | 0.6 |
| PDI | PH | -16.86 | 0.51 |
| EXD | PDI | -16.78 | 0.53 |
| EXD | ITL | -10.68 | 0.73 |
| MH | PH | -8.9 | 0.53 |
| ITC | EXT | -5.91 | 0.68 |
| PDI | MH | -0.39 | 0.53 |
| ICO | IID | -54.57 | 0.53 |
| ICO | PDI | -51.54 | 0.65 |
| ICO | ICR | -30.33 | 0.65 |

***Note.*** BIC = change in Bayesian Information Criterion when that edge is removed from the network. BIC values determine arrow thickness in Supplement Figure S8 and indicate its importance to the network structure, whereby negative values indicate improved model fit if the arrow is added. Directional probability values determine arrow thickness in Figure 3, reflecting the ratio of the arrow being present in that direction in the 10,000 bootstrapped networks compared to the other direction.
