# Supplementary material for: A biopsychological network approach to variables contributing to preoperative quality of life in patients undergoing cardiac surgery
Source: Sci Rep. 2025 Mar 13;15:8746. doi: 10.1038/s41598-025-93467-7 (PMC11906646; doi:10.1038/s41598-025-93467-7)
Supplement: Supplementary file 9 — Supplementary Material 9 [file 41598_2025_93467_MOESM9_ESM.docx]

**Additional figure legends**

**Fig. S1 A.** Raincloud plot showing the data distribution after standardization.

**Fig. S1 B.** Raincloud plot showing the data distribution after nonparanormal transformation.

***Note***. BMI = Body Mass Index, CRP = C-reactive protein, EUR = EuroSCORE II - Risk of 30-day mortality, EXC = Illness Perception Expectations: Consequences, EXD = Pain Disability Index: Expectations (sum), EXP = Illness Perception Expectations: Personal Control, EXT = Illness Perception Expectations: Treatment Control, ICH = Illness Perception: Coherence (Item 7 BIPQ), ICO = Illness Perception: Consequences (Item 1 BIPQ), ICR = Illness Perception: Concern & Emotional Response (Item 6 + 8, BIPQ), IID = Illness Perception: Identity (Item 5 BIPQ), IPC = Illness Perception: Personal Control (Item 3 BIPQ), ITC = Illness Perception: Treatment Control (Item 4 BIPQ), ITL = Illness Perception: Timeline (Item 2 BIPQ), MH = Short Form Health: Current Mental Quality of Life (sum), PDI = Pain Disability Index: Current Disability (sum), PH = Short Form Health: Current Physical Quality of Life (sum).

**Fig. 2.** Pearson correlation plot of raw data before nonparanormal transformation and item indexing.

***Note***. BMI = Body Mass Index, CRP = C-reactive protein, EUR = EuroSCORE II - Risk of 30-day mortality, EXC = Illness Perception Expectations: Consequences, EXD = Pain Disability Index: Expectations (sum), EXP = Illness Perception Expectations: Personal Control, EXT = Illness Perception Expectations: Treatment Control, ICH = Illness Perception: Coherence (Item 7 BIPQ), ICO = Illness Perception: Consequences (Item 1 BIPQ), ICR = Illness Perception: Concern & Emotional Response (Item 6 + 8, BIPQ), IID = Illness Perception: Identity (Item 5 BIPQ), IPC = Illness Perception: Personal Control (Item 3 BIPQ), ITC = Illness Perception: Treatment Control (Item 4 BIPQ), ITL = Illness Perception: Timeline (Item 2 BIPQ), MH = Short Form Health: Current Mental Quality of Life (sum), PDI = Pain Disability Index: Current Disability (sum), PH = Short Form Health: Current Physical Quality of Life (sum).

**Fig. S3.** Cross-sectional network of raw data before nonparanormal transformation and item indexing.

***Note***. BMI = Body Mass Index, CRP = C-reactive protein, EUR = EuroSCORE II - Risk of 30-day mortality, EXC = Illness Perception Expectations: Consequences, EXD = Pain Disability Index: Expectations (sum), EXP = Illness Perception Expectations: Personal Control, EXT = Illness Perception Expectations: Treatment Control, ICH = Illness Perception: Coherence (Item 7 BIPQ), ICO = Illness Perception: Consequences (Item 1 BIPQ), ICR = Illness Perception: Concern & Emotional Response (Item 6 + 8, BIPQ), IID = Illness Perception: Identity (Item 5 BIPQ), IPC = Illness Perception: Personal Control (Item 3 BIPQ), ITC = Illness Perception: Treatment Control (Item 4 BIPQ), ITL = Illness Perception: Timeline (Item 2 BIPQ), MH = Short Form Health: Current Mental Quality of Life (sum), PDI = Pain Disability Index: Current Disability (sum), PH = Short Form Health: Current Physical Quality of Life (sum).

**Fig. S4.** Stability of Expected Influence.

***Note.*** This figure shows the results of the case-dropping bootstrap procedure with 5000 samples drawn. The line depicts the average correlation between the expected influence indices of networks sampled with different proportions of subjects dropped and the original sample. The areas around the line show the range from the 2.5th to the 97.5th quantile.

**Fig. S5.** Stability of Bridge Expected Influence indices.

***Note.*** This figure shows the results of the case-dropping bootstrap procedure with 5000 samples drawn. The line depicts the average correlation between the bridge expected influence indices of networks sampled with different proportions of subjects dropped and the original sample. The areas around the line show the range from the 2.5th to the 97.5th quantile.

**Table T1.** Weight matrix of Gaussian Graphical Model of raw data after nonparanormal transformation and item indexing

***Note.*** Cells contain standardized edge weights (partial correlations).

**Table T2.** Bayesian Information Criterion (BIC) and Directional Probabilities Values of the Arrows in the Directed Acyclic Graph (DAG).

***Note.*** BIC = change in Bayesian Information Criterion when that edge is removed from the network. BIC values determine arrow thickness in Supplement Figure S8 and indicate its importance to the network structure, whereby negative values indicate improved model fit if the arrow is added. Directional probability values determine arrow thickness in Figure 3, reflecting the ratio of the arrow being present in that direction in the 10,000 bootstrapped networks compared to the other direction.
